# AEROS: AdaptivE RObust Least-Squares for Graph-Based SLAM

**DOI:** 10.3389/frobt.2022.789444

**Published:** 2022-04-01

**Authors:** Milad Ramezani, Matias Mattamala, Maurice Fallon

**Affiliations:** ^1^ Robotics and Autonomous Systems Group, DATA61, CSIRO, Brisbane, QLD, Australia; ^2^ Dynamic Robot Systems, Oxford Robotics Institute, Department of Engineering Science, University of Oxford, Oxford, United Kingdom

**Keywords:** robust cost function, outlier resilience, back-end optimisation, factor graph, least squares minimisation, SLAM, perception

## Abstract

In robot localisation and mapping, outliers are unavoidable when loop-closure measurements are taken into account. A single false-positive loop-closure can have a very negative impact on SLAM problems causing an inferior trajectory to be produced or even for the optimisation to fail entirely. To address this issue, popular existing approaches define a hard switch for each loop-closure constraint. This paper presents AEROS, a novel approach to adaptively solve a robust least-squares minimisation problem by adding just a single extra latent parameter. It can be used in the back-end component of the SLAM system to enable generalised robust cost minimisation by simultaneously estimating the continuous latent parameter along with the set of sensor poses in a single joint optimisation. This leads to a very closely curve fitting on the distribution of the residuals, thereby reducing the effect of outliers. Additionally, we formulate the robust optimisation problem using standard Gaussian factors so that it can be solved by direct application of popular incremental estimation approaches such as iSAM. Experimental results on publicly available synthetic datasets and real LiDAR-SLAM datasets collected from the 2D and 3D LiDAR systems show the competitiveness of our approach with the state-of-the-art techniques and its superiority on real world scenarios.

## 1 Introduction

State-of-the-art SLAM systems commonly use sparse pose graphs which are a topological structure made up of nodes representing the poses of the robot/sensor at certain times, and edges representing constraints between those poses. The constraints are the probabilistic expression of either the relative translation between consecutive poses (from an odometry system) or a loop-closure between non-consecutive poses when an explored area is revisited. These constraints are typically determined using a SLAM front-end that converts the raw sensor measurements into constraints added to the pose graph sequentially. The back-end of the graph-based SLAM system estimates the optimal pose trajectory by solving a non-linear least squares minimisation problem. Packages such as GTSAM (Dellaert, 2012), *g* ^2^ *o* (Kümmerle et al., 2011) and the Ceres solver (Agarwal and Mierle, 2012) have been developed for this back-end optimisation.

Erroneous constraints can occur, particularly when determining loop-closures. False-positive loop-closure constraints can cause the optimiser to converge to an incorrect solution resulting in a twisted trajectory and a corrupted map. This, in turn, affects other downstream components such as path planning, collision avoidance and terrain mapping. Hence, the detection of outliers and the mitigation of their negative impact is an important problem.

One way to do this is to sanitise the constraints in the front-end—with high thresholds needed to establish a loop closure. Outlier rejection can be strengthened by accumulating a belief propagated over several constraints (Cummins and Newman, 2008; Cadena et al., 2012), using statistical hypotheses (Olson, 2009; Olson et al., 2012) or by detecting environmentally degenerate conditions (Zhang et al., 2016; Nobili et al., 2018; Ramezani et al., 2020a). There are limits to this approach, with careful and conservative hand-tuning needed to avoid rejecting true loop closures which ought to be detected.

While the front-end aims to remove ill-conditioned constraints during the data association, it is typically a separate processing unit and revisiting a constraint involves reprocessing raw sensor measurements. The alternative has been to add robustness to the back-end of the SLAM system. Back-end outlier rejection is a more abstract problem which is coupled to the pose-graph optimiser—which is the specific problem at hand.

One approach has been to reduce the effect of outliers with adjustments to the least squares minimiser so that they have minimal effect during optimisation. Promising works such as Switchable Constraints (SC) (Sünderhauf and Protzel, 2012), Dynamic Covariance Scaling (DCS) (Agarwal et al., 2013), Max-Mixture (MM) (Olson and Agarwal, 2013) and Graduated Non-Convexity (GNC) non-minimal solvers (Yang et al., 2020) have been proposed to make parameter estimation robust to outliers, each of which have limitations that we discuss further in Sec. 2. As an example, the SC approach requires an additional variable for every loop-closure added to the pose-graph; it also cannot effectively deactivate outliers when the outlier-to-inlier ratio exceeds a threshold (Sünderhauf, 2012). The GNC-based algorithm relies on a specific M-estimator (Zhang, 1997), meaning that algorithm is not general for different scenarios.

In this paper we present AdaptivE RObust least-Squares–AEROS. Our primary contribution is an adaptive robust cost function which can be used to represent the entire set of M-estimators by estimating a hyper-parameter in a joint optimisation along with the pose parameters. This way, we enable to describe the actual residual distribution in various senarios better than M-estimators, which introduce a fixed kernel, for all cases (Chebrolu et al., 2020).

By leveraging the Black-Rangarajan duality (Black and Rangarajan, 1996) and benefiting from the generalised robust cost function proposed by Barron (Barron, 2019), we demonstrate that the robust cost function can be rewritten in the form of a standard least squares minimisation. We aim to jointly estimate the hyper-parameter with the sensor poses. For this purpose, it is not possible to directly use the Barron’s robust kernel in a least squares minimisation (as proven in (Chebrolu et al., 2020)). As a result we take advantage of the Black-Rangarajan duality to convert the optimization problem into an Iteratively Reweighted Least Squares (IRLS) problem instead.

We test our approach using standard publicly available datasets and real 3D data collected using a LiDAR handheld device in both structured and unstructured environments. The result from our algorithm is then evaluated against ground truth trajectories and compared with other outlier-rejection approaches, namely the SC, DCS, GNC algorithms and one of the M-estimators Geman-McClure (GM). We show that our approach can handle a large outlier-to-inlier ratio which is likely to occur when, for instance, visual information is used for loop-closure detection (Cadena et al., 2016).

The remainder of the paper is organised as follows. Section 2 discusses previous approaches that deal with outliers either in the front-end or in the back-end. Section 3 presents our proposed method for adaptive dynamic pose-graph SLAM—beginning with a brief introduction to Switchable Constraints in Section 3.1 and concluding with our proposed adaptive factors. In Section 4, we evaluate the performance of our proposed approach using publicly available synthetic datasets as well as with real data generated by our LiDAR-SLAM system, before presenting conclusions in Section 5.

## 2 Related Work

Many works have been developed to deal with false positive when closing loops in SLAM. These approaches can be divided into two groups depending on whether they sanitise the constraints in the front end or add optimisation robustness in the back end. This section overviews the body of related work according to that split.

### 2.1 Robust Front-End

Appearance-based place recognition algorithms have been developed to propose loop closures in Visual SLAM (V-SLAM). Visual bag-of-words, originally proposed in (Sivic and Zisserman, 2003), match a compound summary of the image to propose image pairs which ought to correspond to the same place. However, relying only on visual information is challenging due to perceptual aliasing, requiring additional verification steps to confirm a loop closure candidate.

To robustify the appearance-based place recognition against outliers, Cummins and Newman (Cummins and Newman, 2008; Cummins and Newman, 2011) proposed a probabilistic framework for appearance-based navigation called FAB-MAP. Robust data association is achieved by combining information about the current pose as well as probabilistic models of the places. Cadena *et al.* (Cadena et al., 2012) improved the robustness of their appearance-based loop closure system by performing a variety of similarity and temporal consistency checks followed by a Conditional Random Field (CRFs) (Lafferty et al., 2001) verification. Similar approaches have been used for data association of visual (Ramos et al., 2009) or LiDAR measurements (Burgard et al., 2008).

Olson (Olson, 2009) proposed a place recognition method which uses spectrally clustered local matches to achieve global consistency. Local uniqueness guaranteed that the loop closure is unique to a particular neighbourhood, while the global sufficiency verifies the unambiguity of the local match within the positional uncertainty of the robot. Later Olson *et al.* (Olson et al., 2012) proposed a simple loop-closure verification method using a temporary pose graph. It included all the candidate loops and performed a Chi-squared test (aka *χ* ^2^ test) before adding them to the final pose graph to be optimised.

There are also methods that reject unreliable loop closures by analysing the structure of the environment. Zhang *et al.* (Zhang et al., 2016) estimated odometry constraints by determining and separating degenerate dimensions in the state space. In this way, the optimisation was constrained to only the well-conditioned dimensions. Nobili *et al.* (Nobili et al., 2018) predicted the alignment risk of two partly-overlapping laser scans, given the initial poses of the scans from which they are captured. While they introduced a metric to detect degenerate dimensions, this approach was not implemented for loop-closure verification. Since the approach was not suitable for real-time operation due to the exhaustive search needed to find overlapped surfaces, later Ramezani *et al.* (Ramezani et al., 2020a) accelerated this method by using a kd-tree.

As a conclusion, front-end robustness requires application dependent tuning and configuration, affecting its generalisation to different scenarios with different sensor configurations. This makes robust back-end methods more appealing, since they provide a general method to deal with outliers.

### 2.2 Robust Back-End

In contrast to robust front-end methods, in these methods the responsibility for determining the validity of loop closures lies on the SLAM back-end, i.e., the optimiser.

A method which has gained in popularity is Switchable Constraints (SC) proposed by Sünderhauf and Protzel (Sünderhauf and Protzel, 2012). As described in Section 3.1, for every loop closure edge added to the pose graph, a switch variable is added to the optimisation. The switch variables are estimated in a joint optimisation with the poses. The objective function of the associated loop closures will be down-weighted if they are erroneous so as to mitigate the impact of outliers. This approach, however, increases computation complexity since each loop closure constraint is associated with this secondary variable. To avoid these additional switch variables, Agarwal *et al.* (Agarwal et al., 2013) suggested Dynamic Covariance Scaling (DCS) whose central idea is the same as SC. Instead of adding an additional switch variable for every loop closure, a closed-form solution is used to select a switch factor for each loop closure. Since the formulation is proportional to the original error of the loop closure constraint, the information matrix of the loop closure constraints is dynamically reweighted without requiring extra computation.

Olson and Agarwal (Olson and Agarwal, 2013) suggested using multi-modal distributions in the maximum likelihood instead of unimodal Gaussians. With this in mind, they replaced the sum-mixture Gaussian with a max-mixture Gaussian and used this approach to detect uncertain loop closures. By defining two components for each loop closure, i.e., the loop closure being computed from the front-end and a null hypothesis which represents the case in which the loop closure is false, the authors allow the optimiser to select a more plausible state. However, this approach does not guarantee convergence to the global optimum.

Latif *et al.* (Latif et al., 2013) introduced an algorithm, called Realizing, Reversing and Recovering (RRR), which clustered the pose graph edges, i.e. constraints consistent with one another, using the place recognition constraints. They conducted two *χ* ^2^ tests to check the consistency of clusters with each other and with the odometry links. A difference between the RRR algorithm and the two previously mentioned approaches (Agarwal et al., 2013; Olson and Agarwal, 2013) is that RRR makes a binary decision and the suspicious loop closure will be omitted if a statistical criteria is not met. In contrast, the other approaches keep the outlier in the optimisation, but with a minimal weight.

M-estimators (Zhang, 1997) are the standard technique for robust optimisation in robotics. They aim to remove the effect of outliers when fitting a model by replacing the standard square error with a robust function that has the same behaviour in a basin close to zero error but have a lower penalising effect outside the basin. Some M-estimators which are commonly used in robotics include Huber (Huber, 1992), Cauchy (Black and Anandan, 1996) and L1-L2 (Zhang, 1997).

There are two problems with M-estimators: their parameters need to be manually tuned and one M-estimator cannot generalise to all the problems, i.e. the selection of an M-estimator depends on the problem and the data. Resolving these problems was the motivation for Agamennoni *et al.* (Agamennoni et al., 2015). They developed an approach of self-tuning M-estimators in which the tuning parameter of the M-estimator was considered as a variable to be itself estimated in the optimisation problem within a two-step Expectation-Maximisation (EM) procedure in an iterative fashion. Although this approach can tune the parameters of the M-estimator automatically, the optimal choice of M-estimator family was still an expert decision. The authors concluded that Student-*t* M-estimator was a much better fit to the residuals than the other estimators they tested, albeit with testing on a limited number of datasets.

Another approach, which used the EM algorithm, was the work of Chebrolu *et al.* (Chebrolu et al., 2020). Instead of estimating the optimal tuning parameter of a specific M-estimator, the authors estimated the shape parameter of a general robust function, proposed by Barron (Barron, 2019), to fit the best curve to the probability distribution of the residuals. Nonetheless, their algorithm produces a sub-optimal solution because the hyperparameters (either the tuning parameter or the shape parameter) and the main parameters (robot poses) are not jointly estimated.

Recently, Yang *et al.* (Yang et al., 2020) proposed an approach to outlier rejection that used an M-estimator and converted it to a series of outlier processes, described in Section 3.3.2, by leveraging the Black-Rangarajan duality (Black and Rangarajan, 1996). The authors used Graduated Non-Convexity (GNC) to achieve a robust estimation without any initialisation. The key idea of GNC is to start with a convex problem and replace the robust function with a surrogate function governed by a control parameter and to gradually recover the original cost function. Nevertheless, this approach needs to update the hyperparameters, in this case the weights, in an update step similar to the EM algorithm. In addition, the choice of M-estimator was a user decision.

In the same context, Lee *et al.* (Lee et al., 2013) showed that the corresponding weights for the loop closure constraints can be chosen using a Cauchy function so that the optimisation becomes convex. They also reweighted the original residuals in an EM procedure. Carlone *et al.* (Carlone et al., 2014) used an L1 proxy to relax a non-convex L0 optimisation to find the largest subset of coherent and observable measurements. However, as noted by the authors, applying convex relaxation does not guarantee the maximum-cardinality of the subset.

Finally, there have also been efforts that breakdown the distinction between front-end and back-end to jointly attack the problem. For example, Wu and Beltrame (Wu and Beltrame, 2020) presented a cluster-based approach that leverages the spatial clustering technique in (Latif et al., 2013) to group loop closures topologically close to one another. They checked the local consistency of each cluster in the front-end with a *χ* ^2^ test, and then they applied a Cluster-based Penalty Scaling (CPS) along with switchable constraints to achieve global consistency in the back-end. However, their approach requires more computation time than either SC or DCS. This complexity is due to the need to examine each cluster in the front-end and adding the clustered-based penalty to the back-end.

## 3 Dynamic Pose-Graph SLAM

A sparse pose graph typically is made up of odometry and loop-closure (LC) factors. Adding these factors to the graph, we seek a Maximum A Posterior (MAP) estimate of the set of poses X . In a LiDAR-SLAM system, the poses correspond to the sensor poses from which the laser scans were collected. To produce a MAP estimate over the variables $X_{i}$ , the product of all the factors must be maximised:

$$X^{\mathrm{MAP}}=\;\underset{X}{\arg\max}\;\underset{i}{\prod }\underset{\text{Odometry Factor}}{\underset{︸}{\phi_{i}\left(X_{i-1},X_{i}\right)}}\underset{j}{\prod }\underset{\text{LC Factor}}{\underset{︸}{\phi_{j}\left(X_{p_{j}},X_{q_{j}}\right)}},$$ (1)

where the odometry factors are defined between consecutive poses $X_{i}$ and $X_{i-1}$ , and $X_{p_{j}}$ and $X_{q_{j}}$ are the tail and head poses of the *jth* loop closure.

Assuming that error is Gaussian distributed, and using the Negative Log Likelihood (NLL), the optimal variable configuration in (1) can be determined by minimising a nonlinear least squares problem:

$$\begin{array}{c} X^{\mathrm{MAP}}=\underset{X}{\text{argmin}}\underset{i}{\sum }\|f_{i}\left(X_{i},X_{i-1}\right)-y_{i}{\|}_{\Sigma_{i}}^{2}\\ +\underset{j}{\sum }\|f_{j}\left(X_{p_{j}},X_{q_{j}}\right)-y_{j}{\|}_{\Lambda_{j}}^{2}, \end{array}$$ (2)

where *y* and *f* denote the measurements and their corresponding non-linear models. Matrices **Σ** and **Λ** are the covariance matrices corresponding to the odometry factors and loop-closures, respectively.

### 3.1 Least Squares With Switch Variables

An efficient approach when dealing with false-positive loop-closures in the back-end is the approach proposed by Sünderhauf and Protzel (Sünderhauf and Protzel, 2012) in which, for each loop-closure added to the factor graph, a switch variable is added to reweight the cost function:

$$\begin{array}{c} {\left\{X,S\right\}}^{\mathrm{MAP}}=\;\underset{X,S}{\text{argmin}}\underset{i}{\sum }\|f_{i}\left(X_{i},X_{i-1}\right)-y_{i}{\|}_{\Sigma_{i}}^{2}\\ +\underset{j}{\sum }\left(\underset{\text{Switched LC Factor}}{\underset{︸}{w\left(s_{j}\right)\|f_{j}\left(X_{p_{j}},X_{q_{j}}\right)-y_{j}{\|}_{\Lambda_{j}}^{2}}}+\underset{\text{Switch Prior}}{\underset{︸}{\|s_{j}-\lambda {\|}_{\sigma_{j}^{2}}^{2}}}\right), \end{array}$$ (3)

in which, the summations can be interpreted as three independent residuals of the odometry, switched loop-closure and switch prior factors. S is a vector of switch variables, *s*_*j*_, being jointly estimated along with X.w (*s*_*j*_) ∈ [0, 1] denotes the weighting function which can be either a simple linear function, e.g. ω (*s*_*j*_) = *s*_*j*_ or a non-linear function such as sigmoid, i.e. $\omega \left(s_{j}\right)=\frac{1}{\left(1+\exp\left(-s_{j}\right)\right)}$ (Sünderhauf, 2012). The scalar *λ* represents an initial guess of the switch variable around which *s*_*j*_ can be adjusted within a standard deviation, *σ*_*j*_, such that the entire range of *ω* is covered.

As noted in (Sünderhauf and Protzel, 2012), all loop-closures are initially treated as being true-positive, i.e. as inliers, thus the initial guess *λ* must be selected such that the weight *w* tends to 1. In the case of using a linear function, e.g. *ω* (*s*_*j*_) = *s*_*j*_, *λ* should be set to 1 with *σ*_*j*_ = 1. If a sigmoid function is used, the initial value of *s*_*j*_ is selected as 10 with *σ*_*j*_ = 20. This setting guarantees that the range (–5, 5), where *w* varies from 0 to 1, is covered (Sünderhauf and Protzel, 2012).

When using the switchable loop-closure constraints, the switch prior is required because only this term behaves as a penalty to prevent the minimisation from driving switch variables of the inliers to zero. Otherwise, it is the natural behaviour of the minimisation to disable all the loop-closures.

The drawback of switchable constraints is that for each loop-closure constraint, a switch variable must be added to the joint optimisation. As a result, with a large number of switch variables, in addition to the complexity of the SLAM problem increasing, there is no guarantee that the entire outliers are down-weighed as shown in Figure 5. These failure cases globally distort the map when compared to the ground truth. However, the map is still locally consistent useful to a robot operating in the environment (Sünderhauf, 2012).

### 3.2 Least Squares With M-Estimators

Another widely used technique to reduce the impact of false-positive loop closures on the optimisation is M-estimation (Zhang, 1997). Using robust M-estimation, (2) can be rewritten by replacing the quadratic cost function in the loop closure term by a robust function of residuals:

$$\begin{array}{c} X^{\mathrm{MAP}}=\;\underset{X}{\text{argmin}}\underset{i}{\sum }\|f_{i}\left(X_{i},X_{i-1}\right)-y_{i}{\|}_{\Sigma_{i}}^{2}\\ +\underset{j}{\sum }\rho \left(\|f_{j}\left(X_{p_{j}},X_{q_{j}}\right)-y_{j}{\|}_{\Lambda_{j}^{\frac{1}{2}}}\right) \end{array}$$ (4)

here, *ρ* is a symmetric, positive-definite function which is convex around zero and has smaller gradient than the quadratic function when far from the minimum, therefore it is less forgiving about outliers.

Throughout the paper, we assume that odometry constraints are reliable and only loop-closure constraints are susceptible to outliers, thus we define a robust cost function for that term. Defining $\nu_{j}=\|f_{j}\left(X_{p_{j}},X_{q_{j}}\right)-y_{j}{\|}_{\Lambda_{j}^{\frac{1}{2}}}$ , (4) yields:

$$\underset{X}{\text{argmin}}\underset{j}{\sum }\rho \left(\nu_{j}\right)$$ (5)

The M-estimator of X based upon the robust cost function *ρ*(*ν*_*j*_) is the solution of the following equation:

$$\underset{j}{\sum }\psi \left(\nu_{j}\right)\frac{\partial \nu_{j}}{\partial X_{i}}=0,\;\text{for}\;i=1,\ldots ,m,$$ (6)

where, $\psi \left(\nu \right)=\frac{d\rho \left(\nu \right)}{d\nu }$ , known as the influence function, is the derivative of the robust function *ρ* with respect to the residual *ν*. The solution to (6) depends highly on the influence function. To solve the problem in (6), the weight function $\omega \left(\nu \right)=\frac{\psi \left(\nu \right)}{\nu }$ is defined, thus:

$$\underset{j}{\sum }\omega \left(\nu_{j}\right)\nu_{j}\frac{\partial \nu_{j}}{\partial X_{i}}=0,$$ (7)

(7) can be solved using the Iterative Reweighted Least Squares (IRLS) method (Zhang, 1997):

$$\underset{X}{\text{argmin}}\underset{j}{\sum }\omega_{k-1}\left(\nu_{j}\right)\nu_{j}^{2}$$ (8)

where, subscript *k* indicates iteration number and *ω*_*k*−1_ is computed based on the optimal state from the previous iteration (*k* − 1).

### 3.3 Least Squares With Adaptive Robust Cost Function

Both the SC technique and the M-estimators aim to mitigate the effect of outliers by reducing their weight in the optimisation. The former has the advantage of dynamically adjust the loop closures contribution by means of switch variables which are jointly estimated in the optimisation. On the other hand, M-estimators do not introduce any extra variables; however their parameters need to be set beforehand and then fixed during the optimisation, regardless of the number of outliers.

**FIGURE 1:**
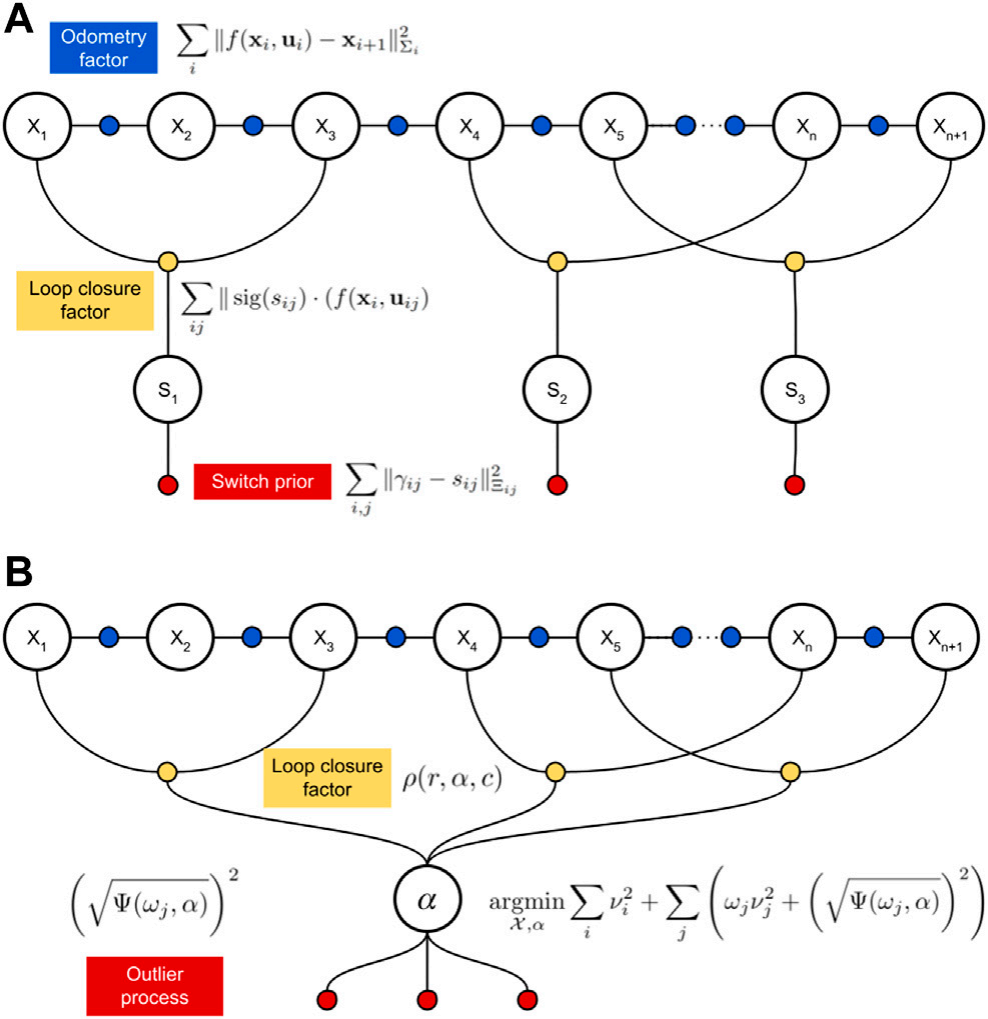
Representation of loop closing factors and their additional components added to the graph by the SC method **(A)** and our proposal **(B)**. As seen, in the SC structure, each switchable loop-closure is accompanied with a switch variable (s_*i*_) and a prior constraint (P_*i*_). In contrast, in the structure of AEROS, all the adaptive loop closures are connected to the latent parameter (*α*) and for every loop closure constraint an outlier process is defined.

Our approach, AEROS, leverages the advantages of the M-estimators for outlier rejection, to avoid adding a switch variable to the pose graph for every loop-closure, while still being able to adapt its behaviour depending on the problem. Figure 1 illustrates the difference between AEROS and the SC approach.

To achieve this behaviour, we utilise the general adaptive robust loss presented by Barron (Barron, 2019), which can represent a wide range of the M-estimators by only changing a parameter. This is, in principle, similar to the approach of Chebrolu *et al.* (Chebrolu et al., 2020), however, we show how to obtain a closed-form expression that allows us to solve the problem in a joint optimisation.

#### 3.3.1 General Adaptive Kernel

The first key part of our approach is the adaptive cost function presented by Barron (Barron, 2019). It can be adjusted to model the behaviour of a large family of robust functions simply by varying a continuous hyper-parameter called the *shape parameter* α∈IR :

$$\rho \left(\nu ,\alpha ,c\right)=\frac{\left|\alpha -2\right|}{\alpha }\left({\left(\frac{{\left(\nu /c\right)}^{2}}{\left|\alpha -2\right|}+1\right)}^{\alpha /2}-1\right),$$ (9)

where, *ν* is the weighted residual between the measurement (*z*) and its fitted value (*y*) represented in (2) and *c* > 0 is a tuning parameter which determines the size of the quadratic basin around *ν* = 0. The tuning parameter *c* needs to be set before optimisation.

**FIGURE 2:**
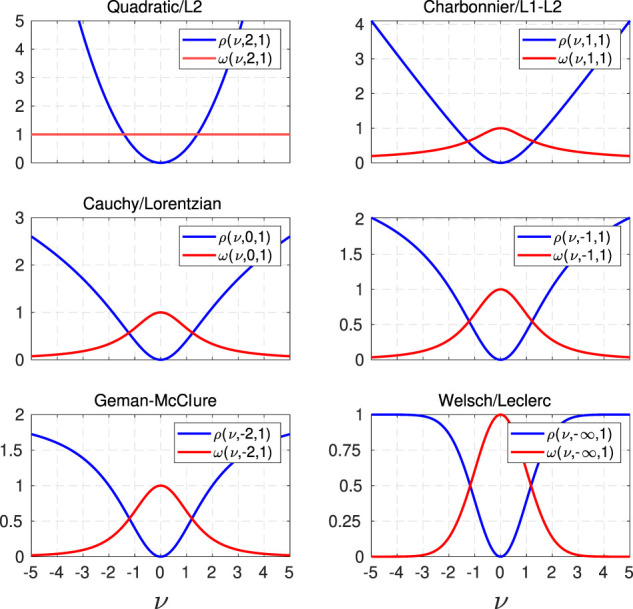
The adaptive loss kernel and its weight for various values of the shape parameter *α*. When *α* is 2, the adaptive loss kernel forms the standard quadratic/L2 function which is not robust to outliers. For *α* equal to 1, 0, -2 and −*∞*, the adaptive loss kernel can form the robust functions known as Charbonnier/L1-L2, Cauchy/Lorentzian, Geman-McClare and Welsh/Leclerc, respectively.

As shown in Figure 2, Barron’s adaptive loss kernel can represent a wide range of well-known robust loss functions for different values of *α*. As discussed in (Barron, 2019), the adaptive kernel is undefined if *α* = {2, 0}. These singularities need to be considered during the optimisation of the shape parameter. However, the adaptive kernel approaches quadratic/L2 loss function or Cauchy kernel (Black and Anandan, 1996) if *α* tends to 2 or 0, respectively. If *α* = 1, the adaptive kernel, $\rho \left(\nu ,1,c\right)=\sqrt{{\left(\nu /c\right)}^{2}+1}-1$ , resembles L2 squared loss near *ν* = 0 and L1 absolute loss when *ν* is large. Due to this behaviour, the robust kernel with *α* = 1 is known as an L1-L2 loss (Zhang, 1997) or a pseudo-Huber since it is a smooth approximation of the Huber loss function (Huber, 1992).

The adaptive loss kernel is presented as:

$$\rho \left(\nu ,\alpha ,c\right)=\left\{\begin{array}{cc} 0.5{\left(\nu /c\right)}^{2}\; & if\alpha \to 2\\ \log\left(0.5{\left(\nu /c\right)}^{2}+1\right)\; & if\alpha \to 0\\ 1-\exp\left(-0.5{\left(\nu /c\right)}^{2}\right)\; & if\alpha \to -\infty \\ \frac{\left|\alpha -2\right|}{\alpha }\left({\left(\frac{{\left(\nu /c\right)}^{2}}{\left|\alpha -2\right|}+1\right)}^{\alpha /2}-1\right)\; & else \end{array}\right.$$ (10)

The weight *ω*(*ν*, *α*, *c*), is the derivative of *ρ* w.r.t. the residual *ν* over the residual, i.e. $\omega =\frac{1}{\nu }\frac{\partial \rho }{\partial \nu }$ , and is defined as:

$$\omega \left(\nu ,\alpha ,c\right)=\left\{\begin{array}{cc} 1/c^{2}\; & if\alpha \to 2\\ 2/\left(\nu^{2}+2c^{2}\right)\; & if\alpha \to 0\\ \frac{1}{c^{2}}\exp\left(-0.5{\left(\nu /c\right)}^{2}\right)\; & if\alpha \to -\infty \\ \frac{1}{c^{2}}{\left(\frac{{\left(\nu /c\right)}^{2}}{\left|\alpha -2\right|}+1\right)}^{\left(\alpha /2-1\right)}\; & else \end{array}\right.$$ (11)

We take advantage of (11) to reweight the loop-closure constraints and to solve the minimisation using the IRLS approach.

#### 3.3.2 Black-Rangarajan Duality

Using the general adaptive kernel, (9), directly in the optimisation problem effectively treats the entire loop closures as outliers by estimating an *α* that downweights all the residuals. To avoid this trivial solution, Barron introduced a partition function, *Z*(*α*), as a penalty. However this makes it impractical to implement the optimisation (please refer to (Barron, 2019) for further details). Instead, Chebrolu *et al.* (Chebrolu et al., 2020) presented an approximation of *Z*(*α*) by means of a truncated partition function which was pre-computed as a look-up table, and used in an Expectation-Maximisation fashion.

In this work, we instead reformulate the problem using the Black-Rangarajan duality (Black and Rangarajan, 1996) to estimate the shape and pose parameters in a joint optimisation. Black and Rangarajan introduced a new variable *ω* ∈ [0, 1] such that the solution of (5) remains unchanged at the minimum. Hence a new objective function is defined as follows:

$$\rho \left(\nu_{j}\right)=\omega_{j}\nu_{j}^{2}+\Psi \left(\omega_{j}\right)$$ (12)

where, Ψ(*ω*) is a penalty term, called an outlier process, whose expression depends on the *ρ*-function. (5) can be redefined as follows:

$$\underset{X,\omega_{j}\in \left[0,1\right]}{\text{argmin}}\underset{j}{\sum }\omega_{j}\nu_{j}^{2}+\Psi \left(\omega_{j}\right)$$ (13)

We aim to achieve the same form as in (5) and (13), thus we use $\omega =\frac{\rho^{\prime }\left(\nu \right)}{2\nu }$ which comes from the differentiation of (5) and (13) with respect to *ν*.

To determine Ψ(*ω*), a function $\phi \left(z\right)=\rho \left(\sqrt{z}\right)$ satisfying lim_*z*→0_ *ϕ*′(*z*) = 1, lim_*z*→*∞*_ *ϕ*′(*z*) = 0 and *ϕ* ^″^(*z*) < 0 is defined (see (Black and Rangarajan, 1996) for more details).

By differentiating (13) with respect to *ω* and replacing *ω* with $\frac{\rho^{\prime }\left(\nu \right)}{2\nu }$ , we achieve:

$$\begin{array}{cc} & \nu^{2}+\Psi^{\prime }\left(\omega \right)=0\\ & \nu^{2}=-\Psi^{\prime }\left(\frac{\rho^{\prime }\left(\nu \right)}{2\nu }\right) \end{array}$$ (14)

Now, by exploiting the function *ϕ* and integrating (14), the outlier process Ψ(*ω*) is obtained:

$$\Psi \left(\omega \right)=\phi \left({\left(\phi^{\prime }\left(\omega \right)\right)}^{-1}\right)-\omega \left({\left(\phi^{\prime }\left(\omega \right)\right)}^{-1}\right)$$ (15)

Using (15), the outlier process corresponding to Barron’s general cost function can be derived as follows:

$$\Psi \left(\omega ,\alpha \right)=\left\{\begin{array}{cc} 0\; & if\alpha \to 2\\ -\log\left(\omega \right)+\omega -1\; & if\alpha \to 0\\ \omega \log\left(\omega \right)-\omega +1\; & if\alpha \to -\infty \\ \frac{\left|\alpha -2\right|}{\alpha }\left(\left(1-\frac{\alpha }{2}\right)\omega^{\frac{\alpha }{\alpha -2}}+\frac{\alpha \omega }{2}-1\right)\; & \alpha <2 \end{array}\right.$$ (16)

As seen in (16), the outlier process is zero for *α* = 2 or not defined for *α* > 2 since there are no robust cost functions with these shape parameters, hence no outlier rejection is processed.

The outlier process in (16) is a tractable, closed-form expression. Then, our formulation for the joint estimation of the latent variable *α* and the parameters X with respect to the odometry factors *i*, loop-closure factors and outlier processes *j* can then be written as:

$$\underset{X,\alpha }{\text{argmin}}\underset{i}{\sum }\nu_{i}^{2}+\underset{j}{\sum }\left(\omega_{j}\nu_{j}^{2}+\Psi \left(\omega_{j},\alpha \right)\right)$$ (17)

In contrast to the SC formulation (3), in the optimisation defined in (17) all of the weights *ω*_*j*_ are a function of the individual parameters *α*. The outlier process term Ψ(*ω*_*j*_, *α*) is not a prior factor but acts as a penalisation for *α* that precludes the optimiser from suppressing all the loop closures.

While (17) now has a tractable expression, it has the disadvantage that the outlier process is not quadratic and thus cannot be used with standard least squares solvers. Fortunately, Rosen *et al.* showed that non-Gaussian factors can still be used if the residuals are always positive and are rewritten as square roots (Rosen et al., 2013), which is true in our case. Hence, the equivalent nonlinear least squares problem becomes:

$$\underset{X,\alpha }{\text{argmin}}\underset{i}{\sum }\nu_{i}^{2}+\underset{j}{\sum }\left(\omega_{j}\nu_{j}^{2}+{\left(\sqrt{\Psi \left(\omega_{j},\alpha \right)}\right)}^{2}\right)$$ (18)

**FIGURE 3:**
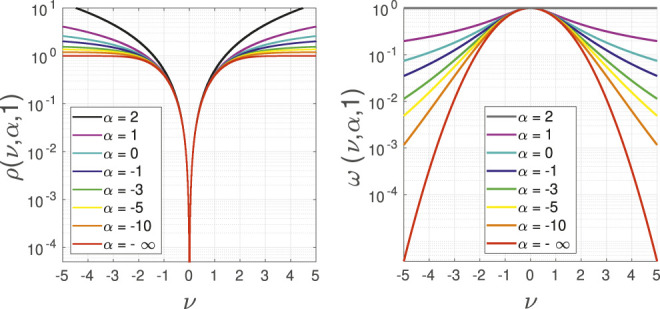
The logarithmic representation of the adaptive loss kernel and its weight function for a multiple values of the shape parameter *α*. It is clearly visible that the difference between alphas smaller than -10 is negligible.

We note that the generalised cost function, as well as its derived weight (in Figure 3), shows negligible change for very small values of *α*, e.g., smaller than -10 in our implementation. Thus, we bound the range of *α* from 2 to 10 with an appropriate variance, e.g., 20, covering this range.

## 4 Experiments

This section details the experiments and evaluations used to compare AEROS to the state-of-the-art methods for outlier rejection, namely SC, DCS, GNC and the Geman-McClure (GM) robust loss. We tested the methods using widely-used public simulated and real datasets, as well as with real data of our own collection. All these datasets have ground truth available and are summarised in Table 1.

**TABLE 1:** 2D and 3D datasets used in our experiments.

Dataset	Number of Poses	Number of Inliers	Outlier Ratio
Manhattan3500	3,500	2099	10, 20, 30, 40 and 50%
(2D, simulated)			
CSAIL	1,045	128	10, 20, 30, 40 and 50%
(2D, simulated)			
INTEL	943	895	10, 20, 30, 40 and 50%
(2D, simulated)			
City10000	10 000	10 688	10%
(2D, simulated)			
Sphere2500	2,500	2,450	10, 20, 30, 40 and 50%
(3D, simulated)			
Newer College	1,147	98	100%
(3D, real data)			

Since we are interested in real-world applications, we implemented an incremental SLAM system and outlier rejection methods using the iSAM2 algorithm as a back-end. We built the system on top of the GTSAM 4.0 library^1^ except the GNC method, for which we used the implementation available in MATLAB R2021a. All approaches were tested on a standard PC with 4 GHz Intel Xeon 4 core processor and 16 GB RAM. Our implementation will be made open source upon publication of this paper.

Regarding the methodology, we ran 10 Monte Carlo simulations for each method on each dataset at different outlier ratios. We corrupted each dataset by adding incorrect loop closures on top of the inliers already available, from 10 to 50% (see Table 1). In this way we aimed to demonstrate the robustness of each method when new incorrect information was added but without removing inliers, which is more likely to occur in real applications.

For the evaluation we used the Absolute Translation Error (ATE) measuring directly the difference between the ground truth and the estimated trajectory. Additionally, to demonstrate the extent to which the negative impact of outliers are attenuated, we used the Cumulative Distribution Function (CDF) to compute the cumulative distribution of errors of each Monte Carlo run.

Lastly, we must mention the parameters used in some methods. For DCS we set the threshold *Φ* = 1 as suggested in (Agarwal et al., 2013). For GNC we used Truncated Least Squares (GNC-TLS) with *TruncationThreshold* = *chi*2*inv* (0.99, 3) = 11.35 in 2D and *TruncationThreshold* = *chi*2*inv* (0.99, 6) = 16.81 in 3D, and *MaxIterations* = 1,000. GNC also requires to choose a local solver, for which we used the *g* ^2^ *o* (Kümmerle et al., 2011) option available in MATLAB.

### 4.1 Synthetic Datasets

Our first experiments considered synthetic datasets commonly used to test SLAM systems: Manhattan3500, City10000 and Sphere2500. We used a version of Manhattan3500 provided by Olson *et al.* (Olson et al., 2006) and the City10000 and Sphere2500 Datasets released by Kaess *et al.* (Kaess et al., 2007) with the iSAM package. As previously mentioned, for each dataset we executed 10 Monte Carlo runs introducing random loop closures with different ratios of outliers. Outliers were generated by randomly selecting two poses and producing a random transform which is supposed to be computed from the alignment of the clouds captured at the selected poses.

We compared the result of our approach with Switchable Constraints (SC) (Sünderhauf and Protzel, 2012), Graduated Non-Convexity (GNC) (Yang et al., 2020), as well as Dynamic Covariance Scaling (DCS) (Agarwal et al., 2013) and the Geman-McClure M-estimator.

**FIGURE 4:**
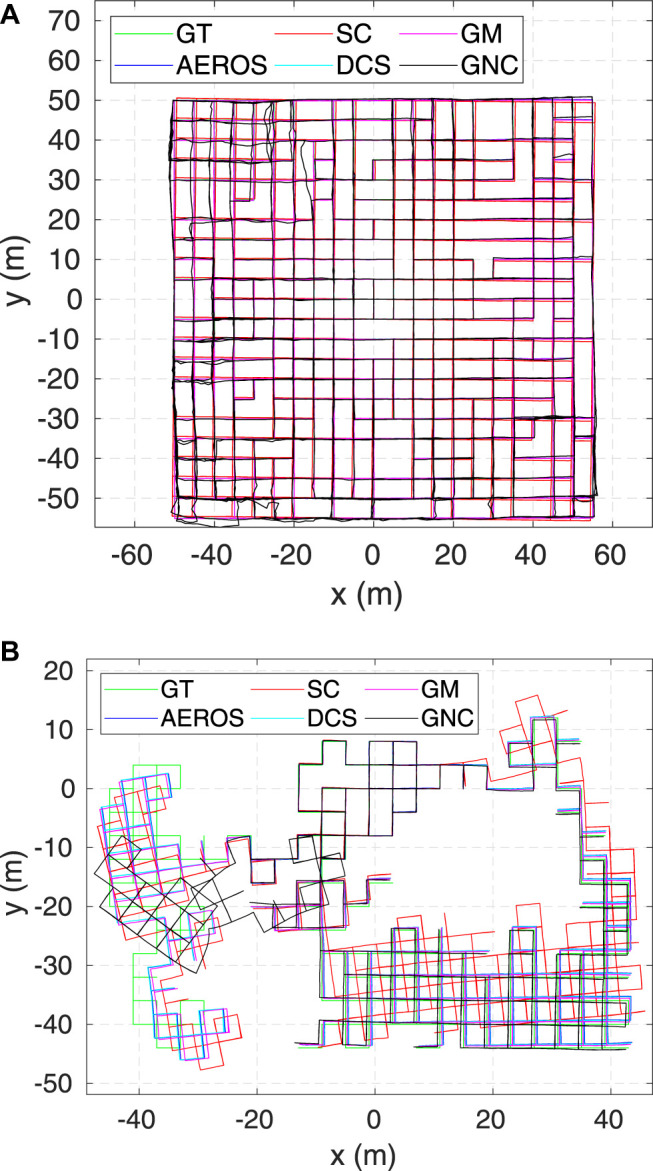
Estimated trajectories for the AEROS algorithm compared to the SC, GM, DCS and GNC solutions as well as the ground truth for the City10000 **(A)** and Manhattan3500 **(B)** each with 10*%* outlier ratio.

**FIGURE 5:**
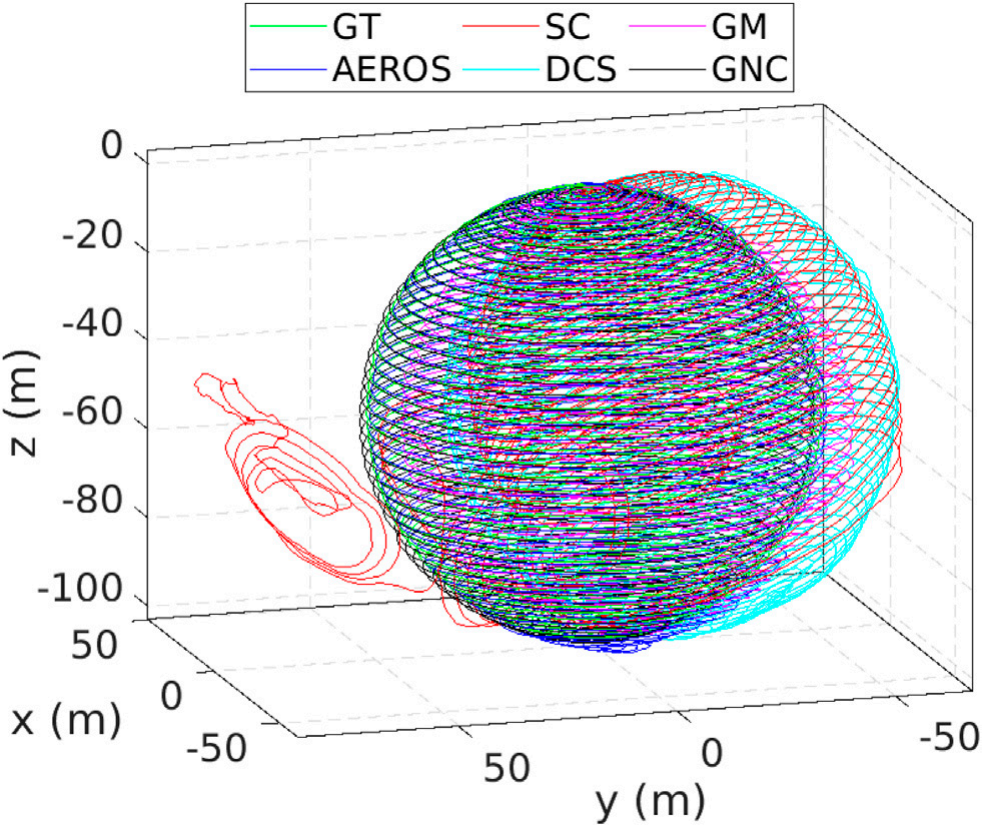
Estimated trajectories compared to the ground truth (green) for the Sphere3500 dataset with 40*%* outlier ratio. As can be seen, the SC approach was unable to reject some of the outliers resulting in global inconsistency. At this Monte-Carlo run, AEROS and GNC outperform the other approaches.

Example trajectories estimated by AEROS and the other approaches are shown in Figure 4 and Figure 5. Our proposed approach shows consistent behaviour with different ratios of outliers. This indicates that once the shape parameter converges, i.e., the optimum robust kernel is obtained, the outliers are then down-weighted and as a result, they have minimal impact on the optimisation.

**FIGURE 6:**
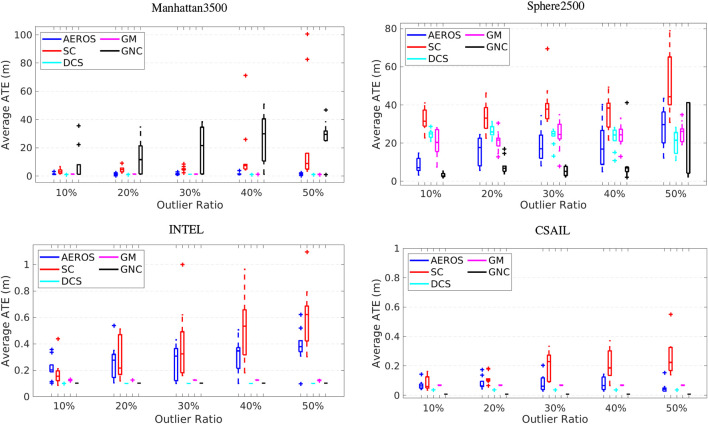
Performance of AEROS compared with the state-of-the-art techniques for increasing outlier ratios. An average trajectory error for (top-left) 2D Manhattan3500 dataset, (top-right) 3D Sphere2500, (bottom-left) 2D INTEL and (bottom-right) 2D CSAIL dataset. Statistics are computed over 10 Monte Carlo runs.

The 2D result of running Manhattan3500 (Figure 6, top-left) shows the stable and consistent performance of the AEROS approach. The 3D result on Sphere2500 (Figure 6, top-right) shows that while a higher outlier ratio affects the AEROS performance, it outperforms the SC algorithm and is competitive with the other approaches.

The performance of the SC approach varies depending on the number of outliers and as reported in (Sünderhauf, 2012), the SC method does not guarantee that the entire false-positive constraints can be detected. The result for Sphere2500 (see Figure 5) demonstrates that some outliers were not detected by the SC approach and consequently they affect the global consistency, although the map is still locally consistent. In addition, the combination of a robust cost function such as Huber with the SC method to resolve global inconsistency, as suggested in (Sünderhauf, 2012), supports the approach we have taken here.

While the DCS and GM methods show stable performance when tested on Manhattan3500 and relatively consistent behaviour on Sphere2500, the GNC approach was unstable on these datasets for different outlier ratios. This is likely to be because the measurements have high covariance so setting the threshold suggested by the $X^{2}$ distribution causes the GNC algorithm to reject even inliers. Nonetheless, the DCS and GM were tested on the same datasets without being affected by this issue.

**FIGURE 7:**
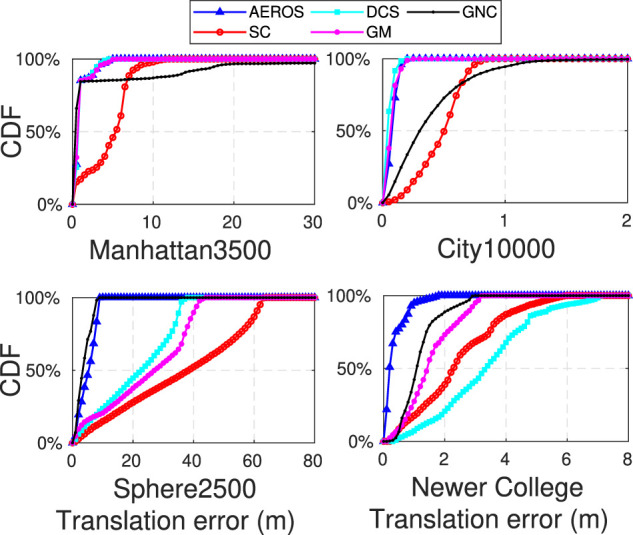
Cumulative distribution of errors for the experiments described in Sec. 4.

So as to quantitatively measure performance of local and global consistency, we computed the cumulative distribution of the pose errors for the algorithms, which are shown in Figure 7. The cumulative distribution of errors for City10000 with 10*%* outlier ratio indicates that all the approaches achieved local consistency. Globally, AEROS, DCS and GM slightly outperform the SC and GNC algorithms due to a rotation in the maps produced by these algorithms. This could have been caused by an outlier that was not properly detected and down-scaled when the outlier ratio was low.

The cumulative distribution of errors for Manhattan3500 also shows that our approach, along with DCS and GM, better maintained global consistency in the less connected regions on the left of the plot compared to the SC and GNC methods.

### 4.2 Real 2D Data

As we are interested in the real-world application of our SLAM system, we also tested AEROS and the other approaches on the CSAIL and INTEL datasets described in (Rosen et al., 2019). However, these datasets do not provide false-positive loop closures, so we randomly added outliers with the same procedure as for the synthetic datasets. Figure 6 (bottom) shows the results for INTEL (left) and CSAIL (right). Although AEROS demonstrates consistent performance with a range of ATE between ∼3 cm to ∼20cm, the DCS, GM and GNC algorithms achieved stable ATE accuracy of ∼4cm, ∼7 cm and ∼1cm, respectively, over CSAIL. The range of ATE for AEROS over the INTEL dataset is 10–60 cm, while for DCS, GM and GNC, ATE is stable at about 10 cm, 12 and 10 cm, respectively. This tolerance in ATE indicates that AEROS was unable to achieve optimal performance due to a variance in the *α* estimate for the same experiment in different Monte-Carlo runs. In other words, a slight deviation in the estimation of *α* can cause a few decimeters variation in ATE. This, however, requires further investigation.

### 4.3 Real 3D Data

To test our algorithm in real scenarios, we used our recently published dataset which is described in (Ramezani et al., 2020b). The dataset was collected using a handheld device consisting of a 10 Hz Ouster OS1-64 LiDAR and a 30 Hz Intel RealSense D435i stereo camera, each of which have a built-in IMU. We used the long experiment (experiment #2) from the dataset. This experiment includes the trajectory stretching from a college quad with well-structured area to a garden area with dense foliage and minimal built structure. This wide variety of environments poses a serious challenge for visual and LiDAR-SLAM systems.

Our LiDAR-SLAM system estimates the motion of the device using Iterative Closest Point (ICP) (Besl and McKay, 1992) at 2 Hz using visual odometry as a motion prior (Ramezani et al., 2020a). To detect loop closures, we use both geometric- and appearance-based means to propose loop-closures which are verified by ICP registration. The geometric method searches for loop closure candidates based on the spatial distance, whereas, our appearance-based method employs a bag-of-words approach (Gálvez-López and Tardos, 2012) using ORB features (Rublee et al., 2011).

**FIGURE 8:**
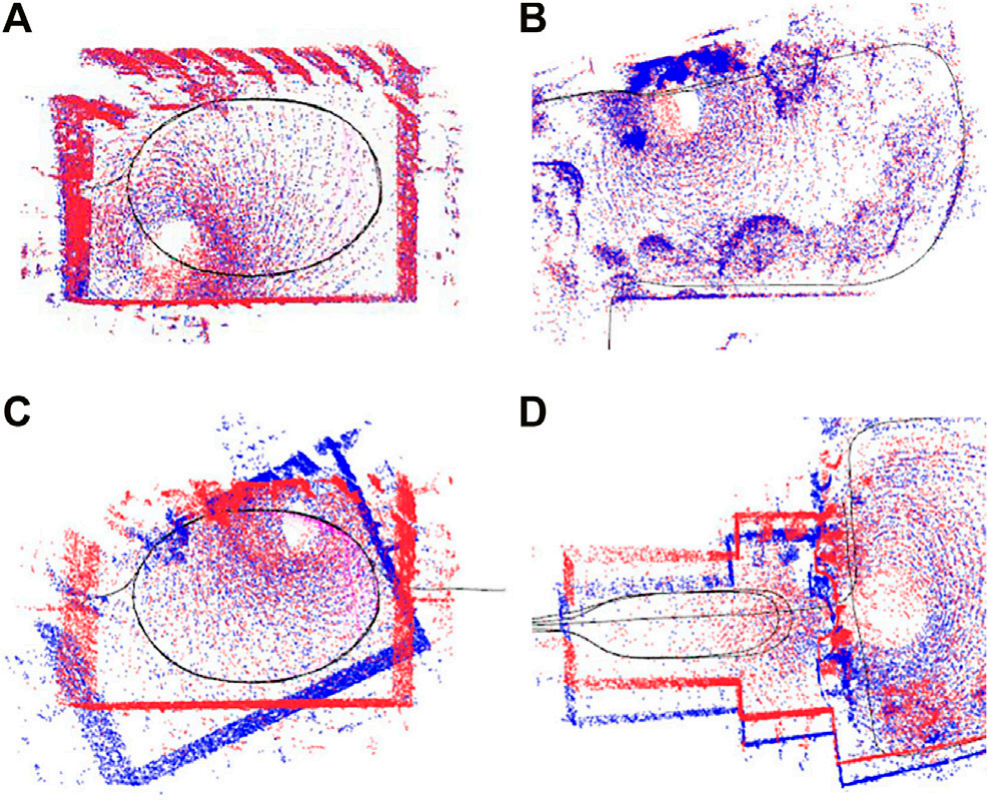
Examples of loop closure constraints created by our (intentionally relaxed) LiDAR-SLAM system. **(A,B)**: True-positive loop closures show the red and blue point clouds are properly aligned. **(C,D)**: False-positive loop closures which do not properly align the red and blue point clouds but are plausible as many points have very low registration error.

Our LiDAR-SLAM system includes a front-end unit to filter loop closures. Once the candidate point clouds are registered by ICP, we analyse the point set (with a maximum of 20 cm point-to-plane error) and determine if there exists points with a diversity of normals, i.e. there is no degeneracy. We accept the loop candidate and it is then added to the pose graph as a true-positive. Figure 8A,B shows some examples of the true-positive loop closures from the quad and parkland area.

As mentioned previously, a key design criterion is that loop closures are readily identified (for proper situational awareness) so it is important not to set thresholds for this module very high. We want to have frequent true-positives yet be robust in the back-end to false-positives.

In order to test the robustness of different algorithms in this dataset, we introduced realistic loop candidates by relaxing our appearance-based loop proposal mechanism. These candidates were generated using visual appearance similarity, which can introduce challenging loop closures to the back-end in scenes with repetitive structure and symmetries, such as the Quad. Having this kind of partly incorrect loop candidates is less likely when randomly generating false-positive loop closures. A few examples of these loop candidates are shown in Figure 8C,D. Note that we raised our front-end loop closure threshold such that it penalised loop candidates and even candidates which may not be entirely wrong such as the one in Figure 8D. Loop closures that were labelled as false-positive were added as outliers to our real dataset.

Figure 10A, shows the ground truth trajectory of the experiment (green), along with the true-positive (blue) and the false-positive (red) loop candidates. It can be seen that the majority of rejected loop closures are in the parkland area (with difficult to distinguish foliage).

**FIGURE 9:**
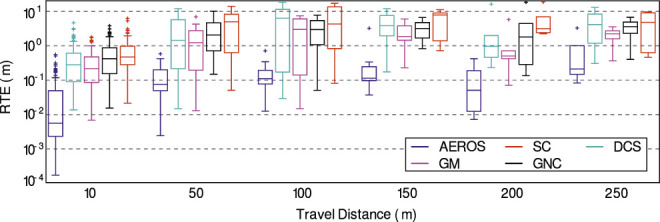
Comparison of AEROS versus the other approaches, DCS, SC, GM and GNC on the NCD real data. Relative Translation Errors (RTE) are measured over various intervals of the trajectory of length 10, 50, 100, 150, 200, 250 m.

**FIGURE 10:**
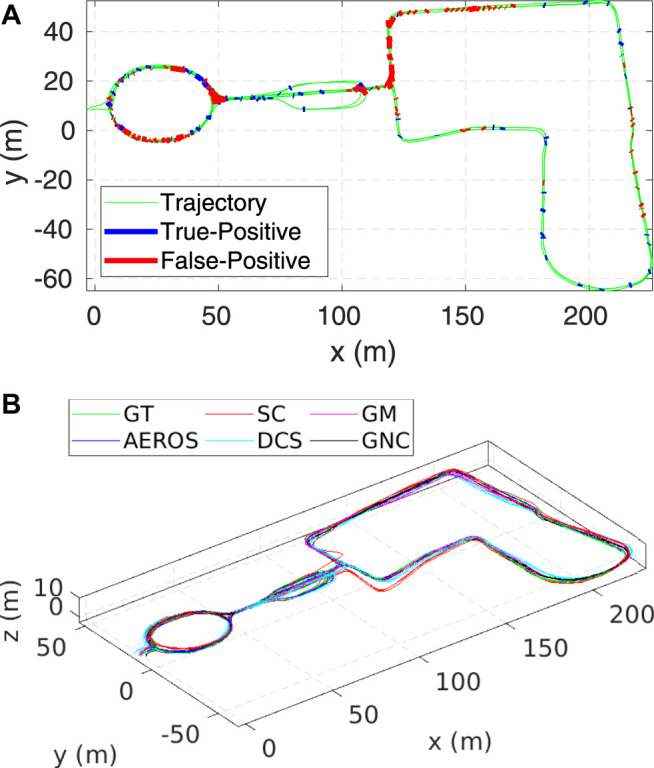
**(A)**: Ground Truth trajectory of the Newer College dataset (the long experiment). The blue and red lines indicate the correct and the incorrect loop closures, respectively. Each line connects two nodes of the pose graph—the head and the tail of the loop closure edge. **(B)**: A 3D view of the trajectory estimated by the AEROS algorithm (blue) versus the SC solution (red), compared to the ground truth (green).

We ran AEROS and the other methods on the dataset and computed the ATE (Figure 7, bottom-right) to measure performance over the entire trajectory. In addition, we computed the Relative Translation Error (RTE) to measure local performance at different scales (Figure 9). This was done by first aligning the estimated trajectories relative to the ground truth using Umeyama alignment (Umeyama, 1991) as implemented in the Python package *evo* (Grupp, 2017). As seen, AEROS outperformed the other approaches, with lower error than the other methods. The reason for this could be attributed to better adaptation of the robust kernels over the residual distribution which can then benefit from partially correct loop closures rather than totally accepting them as inliers or fully rejecting them as outliers. M-estimators have a similar mechanism to down-weight the outliers. However, they present a fixed curve that can totally remove the effect of partially correct loop closures. Figure 10B, shows example trajectories obtained for the different methods tested.

### 4.4 Computation Time

**FIGURE 11:**
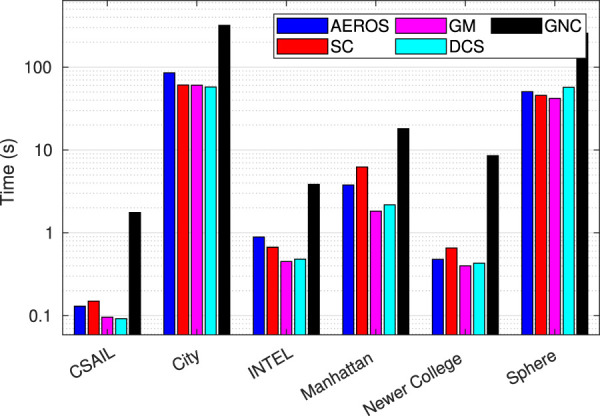
Average computation time for the datasets used in our experiments in Section 4.1, Section 4.2, and Section 4.3.

Figure 11 shows the average computation time over the datasets used in our experiments for a specific outlier to noise ratio.

Both AEROS and SC require more computation time than DCS and GM, which are 10–30*%* faster because they do not introduce any extra variables in the optimisation. Still, we consider AEROS to be competitive considering its adaptive properties, and it is suitable for real-time operation as observed in the Newer College dataset experiments. The GNC approach on the other hand requires more computation time compared to the other techniques. As mentioned before, we used a MATLAB implementation of the GNC algorithm, which could affect the run-time, while the other approaches were implemented in C++. Furthermore we used *g* ^2^ *o* as a solver in the GNC implementation while iSAM was used for the implementation of the other approaches.

### 4.5 Analysis of the Shape Parameter

**FIGURE 12:**
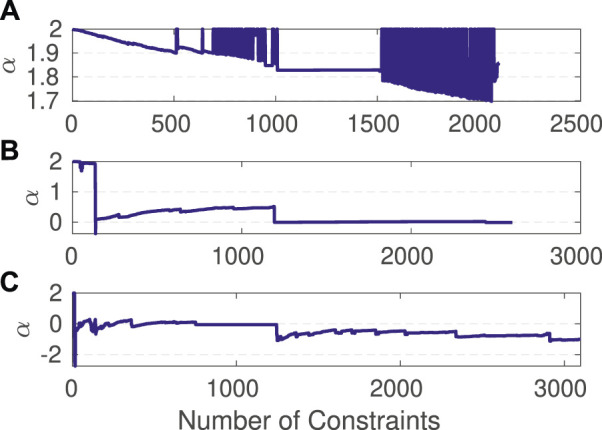
Estimation of the shape parameter over the Manhattan3500 dataset with zero **(A)**, 500 **(B)** and 1,000 **(C)** outliers.

As previously noted, the main advantages of AEROS are due to the optimisation of the shape parameter *α*, which allows us to cover a wide range of M-Estimators. To investigate the convergence of *α*, we used the Manhattan3500 dataset with a different numbers of random outliers. As shown in Figure 12A, without the outliers, *α* stays relatively close to 2, indicating that the adaptive cost function behaves the same as the standard quadratic function. By increasing the number of outliers to 500 (Figure 12B), the shape parameter converged to zero, meaning that the adaptive cost function shows a similar behaviour as the Cauchy fixed kernel. Lastly, we added 1,000 outliers to the dataset (Figure 12C) and *α* gradually converged to −1, for which it is expected to behave similarly to the GM kernel.

## 5 Conclusion and Future Work

In this paper, we introduced a novel approach to deal with false-positive loop closures in LiDAR-SLAM based on the adaptive loss function presented by Barron (Barron, 2019). This adaptive loss function is able to represent a wide range of M-Estimators by tuning a single latent variable. While we formulated the pose-graph SLAM problem as a least squares minimisation, as is typical, we showed how to reformulate it to jointly estimate the poses and the latent parameter. This formulation allowed us to detect and down-weight false-positive loop closures without requiring an extra variable per loop-closure in the optimisation.

We examined our approach with experiments from standard synthetic datasets, as well as real 2D and a large-scale 3D outdoor LiDAR dataset. Experimental results demonstrated that our approach outperforms the Switchable Constraints technique (Sünderhauf and Protzel, 2012) in both 2D and 3D scenarios, and is competitive to other approaches such as DCS while being intrinsically adaptive. In future work we aim to investigate further the properties of the adaptive loss function in real applications, looking towards the development of adaptive real-time algorithms.

## Data Availability

The original contributions presented in the study are included in the article/Supplementary Material, further inquiries can be directed to the corresponding author.

## Footnotes

1
https://github.com/borglab/gtsam

## References

[B1] Agamennoni G. Furgale P. Siegwart R. (2015). “Self-tuning M-Estimators,” in 2015 IEEE International Conference on Robotics and Automation (ICRA), 4628–4635. 10.1109/icra.2015.7139840

[B2] Agarwal P. Tipaldi G. D. Spinello L. Stachniss C. Burgard W. (2013). “Robust Map Optimization Using Dynamic Covariance Scaling,” in 2013 IEEE International Conference on Robotics and Automation, 62–69. 10.1109/icra.2013.6630557

[B3] Agarwal S. Mierle K. (2012). Ceres Solver.

[B4] Barron J. T. (2019). “A General and Adaptive Robust Loss Function,” in The IEEE Conference on Computer Vision and Pattern Recognition (CVPR). 10.1109/cvpr.2019.00446

[B5] Besl P. J. McKay N. D. (1992). “Method for Registration of 3-D Shapes,” in Sensor Fusion IV: Control Paradigms and Data Structures (International Society for Optics and Photonics), 1611, 586–606.

[B6] Black M. J. Anandan P. (1996). The Robust Estimation of Multiple Motions: Parametric and Piecewise-Smooth Flow Fields. Computer Vis. Image Understanding 63, 75–104. 10.1006/cviu.1996.0006

[B7] Black M. J. Rangarajan A. (1996). On the Unification of Line Processes, Outlier Rejection, and Robust Statistics with Applications in Early Vision. Int. J. Comput. Vis. 19, 57–91. 10.1007/bf00131148

[B8] Burgard W. Brock O. Stachniss C. (2008). CRF-matching: Conditional Random Fields for Feature-Based Scan Matching.

[B9] Cadena C. Carlone L. Carrillo H. Latif Y. Scaramuzza D. Neira J. (2016). Past, Present, and Future of Simultaneous Localization and Mapping: Toward the Robust-Perception Age. IEEE Trans. Robot. 32, 1309–1332. 10.1109/tro.2016.2624754

[B10] Cadena C. Galvez-López D. Tardós J. D. Neira J. (2012). Robust Place Recognition with Stereo Sequences. IEEE Trans. Robot. 28, 871–885. 10.1109/tro.2012.2189497

[B11] Carlone L. Censi A. Dellaert F. (2014). “Selecting Good Measurements via L1 Relaxation: A Convex Approach for Robust Estimation over Graphs,” in 2014 IEEE/RSJ International Conference on Intelligent Robots and Systems (IEEE), 2667–2674.

[B12] Chebrolu N. Läbe T. Vysotska O. Behley J. Stachniss C. (2020). Adaptive Robust Kernels for Non-linear Least Squares Problems. arXiv preprint arXiv:2004.14938.

[B13] Cummins M. Newman P. (2011). Appearance-only SLAM at Large Scale with FAB-MAP 2.0. Int. J. Robotics Res. 30, 1100–1123. 10.1177/0278364910385483

[B14] Cummins M. Newman P. (2008). FAB-MAP: Probabilistic Localization and Mapping in the Space of Appearance. Int. J. Robotics Res. 27, 647–665. 10.1177/0278364908090961

[B15] Dellaert F. (2012). “Factor Graphs and GTSAM: A Hands-On Introduction,” in Tech. Rep. (Georgia Institute of Technology).

[B16] Galvez-López D. Tardos J. D. (2012). Bags of Binary Words for Fast Place Recognition in Image Sequences. IEEE Trans. Robot. 28, 1188–1197. 10.1109/tro.2012.2197158

[B17] Grupp M. (2017). Evo: Python Package for the Evaluation of Odometry and Slam. Dataset Available at: https://github.com/MichaelGrupp/evo .

[B18] Huber P. J. (1992). “Robust Estimation of a Location Parameter,” in Breakthroughs in Statistics (Springer), 492–518. 10.1007/978-1-4612-4380-9_35

[B19] Kaess M. Ranganathan A. Dellaert F. (2007). “iSAM: Fast Incremental Smoothing and Mapping with Efficient Data Association,” in Proceedings 2007 IEEE International Conference on Robotics and Automation (IEEE), 1670–1677. 10.1109/robot.2007.363563

[B20] Kümmerle R. Grisetti G. Strasdat H. Konolige K. Burgard W. (2011). “g^2^o: A General Framework for Graph Optimization,” in 2011 IEEE International Conference on Robotics and Automation, 3607–3613. 10.1109/icra.2011.5979949

[B21] Lafferty J. McCallum A. Pereira F. C. (2001). Conditional Random Fields: Probabilistic Models for Segmenting and Labeling Sequence Data.

[B22] Latif Y. Cadena C. Neira J. (2013). Robust Loop Closing over Time for Pose Graph SLAM. Int. J. Robotics Res. 32, 1611–1626. 10.1177/0278364913498910

[B23] Lee G. H. Fraundorfer F. Pollefeys M. (2013). “Robust Pose-Graph Loop-Closures with Expectation-Maximization,” in 2013 IEEE/RSJ International Conference on Intelligent Robots and Systems (IEEE), 556–563. 10.1109/iros.2013.6696406

[B24] Nobili S. Tinchev G. Fallon M. (2018). “Predicting Alignment Risk to Prevent Localization Failure,” in 2018 IEEE International Conference on Robotics and Automation (ICRA), 1003–1010. 10.1109/icra.2018.8462890

[B25] Olson E. Agarwal P. (2013). Inference on Networks of Mixtures for Robust Robot Mapping. Int. J. Robotics Res. 32, 826–840. 10.1177/0278364913479413

[B26] Olson E. Leonard J. Teller S. (2006). “Fast Iterative Alignment of Pose Graphs with Poor Initial Estimates,” in Proceedings 2006 IEEE International Conference on Robotics and Automation, 2006 (ICRA 2006IEEE), 2262–2269.

[B27] Olson E. (2009). Recognizing Places Using Spectrally Clustered Local Matches. Robotics Autonomous Syst. 57, 1157–1172. 10.1016/j.robot.2009.07.021

[B28] Olson E. Strom J. Morton R. Richardson A. Ranganathan P. Goeddel R. (2012). Progress toward Multi-Robot Reconnaissance and the MAGIC 2010 Competition. J. Field Robotics 29, 762–792. 10.1002/rob.21426

[B29] Ramezani M. Tinchev G. Iuganov E. Fallon M. (2020a). Online LiDAR-SLAM for Legged Robots with Robust Registration and Deep-Learned Loop Closure. arXiv preprint arXiv:2001.10249.

[B30] Ramezani M. Wang Y. Camurri M. Wisth D. Mattamala M. Fallon M. (2020b). The Newer College Dataset: Handheld LiDAR, Inertial and Vision with Ground Truth. arXiv preprint arXiv:2003.05691.

[B31] Ramos F. Kadous M. W. Fox D. (2009). “Learning to Associate Image Features with CRF-Matching,” in Experimental Robotics (Springer), 505–514. 10.1007/978-3-642-00196-3_58

[B32] Rosen D. M. Carlone L. Bandeira A. S. Leonard J. J. (2019). Se-sync: A Certifiably Correct Algorithm for Synchronization over the Special Euclidean Group. Int. J. Robotics Res. 38, 95–125. 10.1177/0278364918784361

[B33] Rosen D. M. Kaess M. Leonard J. J. (2013). “Robust Incremental Online Inference over Sparse Factor Graphs: Beyond the Gaussian Case,” in 2013 IEEE International Conference on Robotics and Automation (IEEE), 1025–1032. 10.1109/icra.2013.6630699

[B34] Rublee E. Rabaud V. Konolige K. Bradski G. (2011). “ORB: An Efficient Alternative to SIFT or SURF,” in 2011 International conference on computer vision (IEEE), 2564–2571. 10.1109/iccv.2011.6126544

[B35] Sivic J. Zisserman A. (2003). “Video Google: A Text Retrieval Approach to Object Matching in Videos,” in Null (IEEE), 1470. 10.1109/iccv.2003.1238663

[B36] Sünderhauf N. Protzel P. (2012). “Towards a Robust Back-End for Pose Graph SLAM,” in 2012 IEEE International Conference on Robotics and Automation, 1254–1261. 10.1109/icra.2012.6224709

[B37] Sünderhauf N. (2012). Robust Optimization for Simultaneous Localization and Mapping. Ph.D. thesisTechnischen Universitat Chemnitz.

[B38] Umeyama S. (1991). Least-squares Estimation of Transformation Parameters between Two point Patterns. IEEE Trans. Pattern Anal. Machine Intell. 13, 376–380. 10.1109/34.88573

[B39] Wu F. Beltrame G. (2020). Cluster-based Penalty Scaling for Robust Pose Graph Optimization. IEEE Robotics Automation Lett. 5 (4), 6193–6200. 10.1109/lra.2020.3011394

[B40] Yang H. Antonante P. Tzoumas V. Carlone L. (2020). Graduated Non-convexity for Robust Spatial Perception: From Non-minimal Solvers to Global Outlier Rejection. IEEE Robot. Autom. Lett. 5, 1127–1134. 10.1109/lra.2020.2965893

[B41] Zhang J. Kaess M. Singh S. (2016). “On Degeneracy of Optimization-Based State Estimation Problems,” in 2016 IEEE International Conference on Robotics and Automation (ICRA), 809–816. 10.1109/icra.2016.7487211

[B42] Zhang Z. (1997). Parameter Estimation Techniques: A Tutorial with Application to Conic Fitting. Image Vis. Comput. 15, 59–76. 10.1016/s0262-8856(96)01112-2

