# Supplementary material for: AEROS: AdaptivE RObust Least-Squares for Graph-Based SLAM
Source: Front Robot AI. 2022 Apr 1;9:789444. doi: 10.3389/frobt.2022.789444 (PMC9010720; doi:10.3389/frobt.2022.789444)
Supplement: Supplementary file 1 [file DataSheet1.pdf]

Supplementary Material

APPENDICES

Practical Alternative to Barron's Cost Function and its Gradient and Ψ-Function

As noted in (Barron, 2019), because of the existence of singularities ($\alpha = 0$ or 2) in the general robust kernel and instability of the kernel close to singular values, we use the following equations in our implementation to guard against indeterminacy when α converges to the singularities.

$$\rho(\nu, \alpha, c) = \frac{p}{q}\left(\left(\frac{(\nu/c)^2}{p} + 1\right)^{q/2} - 1\right), \tag{S1}$$

$$\omega(\nu, \alpha, c) = \frac{1}{c^2}\left(\frac{(\nu/c)^2}{p} + 1\right)^{q/2-1}, \tag{S2}$$

Accordingly, the Ψ-function is modified as:

$$\Psi(\omega, \alpha) = \begin{cases} 0 & \text{if } \alpha \to 2 \\ \frac{p}{q}\left((1 - \frac{q}{2})\omega^{\frac{q}{q-2}} + \frac{q\omega}{2} - 1\right) & \text{if } \alpha < 2 \end{cases} \tag{S3}$$

where, $p = |\alpha - 2| + \zeta$ and $\zeta = 10^{-5}$. Depending on whether α is positive or negative, q is defined as:

$$q = \begin{cases} \alpha + \zeta & \text{if } \alpha \geq 0 \\ \alpha - \zeta & \text{if } \alpha < 0 \end{cases} \tag{S4}$$

Maximum Likelihood Least Squares Minimisation for Outlier Processes

According to (Rosen et al., 2013), supposing that $f : \Omega \to \mathbb{R}$ is a function with the corresponding factors:

$$f(\alpha) = \prod_i f_i(\alpha), \tag{S5}$$

If $f_i(\alpha) > 0$ and $\|f_i\|_\infty < c_i$, where c_i is a constant $c_i \in \mathbb{R}$, we can define:

$$\begin{aligned} \nu_i &: \Omega \to \mathbb{R} \\ \nu_i(\alpha) &= \sqrt{\ln c_i - \ln f_i(\alpha)} \end{aligned} \tag{S6}$$

Then the Maximum Likelihood solution can be estimated using Least Squares Minimisation:

$$\operatorname*{argmax}_{\alpha \in \Omega} f(\alpha) = \operatorname*{argmin}_{\alpha \in \Omega} \sum_i \nu_i^2(\alpha) \tag{S7}$$

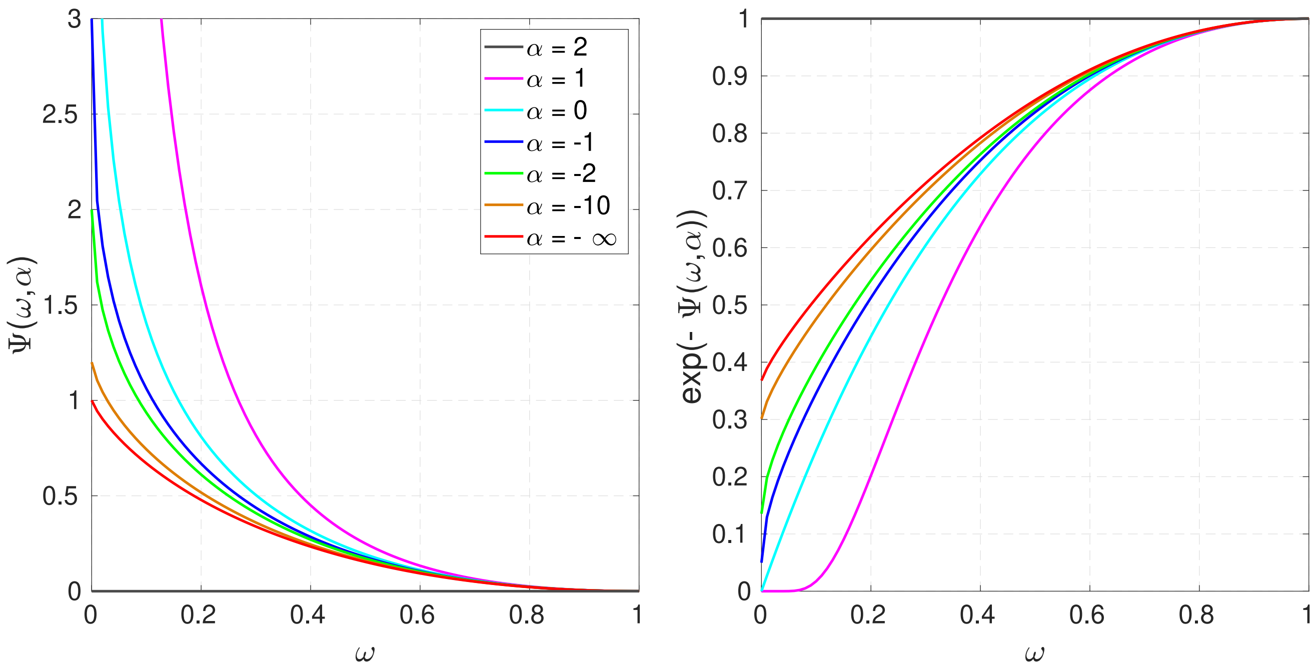

Figure S1. The outlier process for several values of α (left) and the corresponding exponential function of the negative outlier process (right).

To obtain (18), Please see the paper, we consider $f_i = \exp\big(-\Psi(\omega_i, \alpha)\big)$ so that $0 < f_i < 1$. This is true because Ψ is defined in $[0, +\infty)$ (see Fig. S1). Given $c_i = 1$, $\nu_i(\omega_i, \alpha)$ can be defined as:

$$\nu_i(\omega_i, \alpha) = \sqrt{\Psi(\omega_i, \alpha)} \tag{S8}$$

Finally, (S8) can be used as the standard square residual in the least squares minimisation expressed in (18).

REFERENCES

Barron, J. T. (2019). A General and Adaptive Robust Loss Function. In *The IEEE Conference on Computer Vision and Pattern Recognition (CVPR)*

Rosen, D. M., Kaess, M., and Leonard, J. J. (2013). Robust incremental online inference over sparse factor graphs: Beyond the Gaussian case. In *2013 IEEE International Conference on Robotics and Automation* (IEEE), 1025–1032
